# Generation and characterization of an *Il2rg* knockout Syrian hamster model for XSCID and HAdV-C6 infection in immunocompromised patients

**DOI:** 10.1242/dmm.044602

**Published:** 2020-08-27

**Authors:** Rong Li, Baoling Ying, Yanan Liu, Jacqueline F. Spencer, Jinxin Miao, Ann E. Tollefson, James D. Brien, Yaohe Wang, William S. M. Wold, Zhongde Wang, Karoly Toth

**Affiliations:** 1 Department of Animal, Dairy, and Veterinary Sciences, Utah State University, Logan, UT 84322, USA; 2 Department of Molecular Microbiology and Immunology, Saint Louis University School of Medicine, St Louis, MO 63104, USA; 3 National Center for International Research in Cell and Gene Therapy, School of Basic Medical Sciences, Academy of Medical Sciences, Zhengzhou University, Zhengzhou 450052, China; 4 Centre for Biomarkers & Biotherapeutics, Barts Cancer Institute, Queen Mary University of London, London EC1M 6BQ, UK

**Keywords:** CRISPR, Syrian hamster, Adenovirus, Animal model, Knockout

## Abstract

Model animals are indispensable for the study of human diseases, and in general, of complex biological processes. The Syrian hamster is an important model animal for infectious diseases, behavioral science and metabolic science, for which more experimental tools are becoming available. Here, we describe the generation and characterization of an interleukin-2 receptor subunit gamma (*Il2rg*) knockout (KO) Syrian hamster strain. In humans, mutations in *IL2RG* can result in a total failure of T and natural killer (NK) lymphocyte development and nonfunctional B lymphocytes (X-linked severe combined immunodeficiency; XSCID). Therefore, we sought to develop a non-murine model to study XSCID and the infectious diseases associated with IL2RG deficiency. We demonstrated that the *Il2rg* KO hamsters have a lymphoid compartment that is greatly reduced in size and diversity, and is impaired in function. As a result of the defective adaptive immune response, *Il2rg* KO hamsters developed a more severe human adenovirus infection and cleared virus less efficiently than immune competent wild-type hamsters. Because of this enhanced virus replication, *Il2rg* KO hamsters developed more severe adenovirus-induced liver pathology than wild-type hamsters. This novel hamster strain will provide researchers with a new tool to investigate human XSCID and its related infections.

## Abstract (precis)

**Summary:** Syrian hamsters are an important rodent species for studying multiple human diseases. We describe the generation and characterization of a hamster strain that is defective in the XCSID-associated *IL2RG* gene.

## INTRODUCTION

Although advancements have been made in the application of *in vitro* and *in silico* systems, the complexity of most diseases can be captured only in animal models. For animal experiments, the preferred taxonomic group is rodents; the biology of these animals is sufficiently similar to that of humans, their husbandry is relatively easy and cheap, and there are fewer regulatory requirements associated with their use. The Syrian hamster (*Mesocricetus auratus*) is the model of choice in certain fields of medical science. The husbandry and handling of hamsters is easy (they do not jump and hardly bite), they reproduce faster than mice (hamster gestation period is 16 days and average litter size is 8-12) and are available from commercial suppliers. Hamsters have long been used in behavioral science, especially to study the effects of seasonal endocrinological changes and the circadian rhythm (De Lorme et al., 2013; Harris, 2017; Korf, 2018; Loudon et al., 2007). They are also useful for research in the fields of reproductive biology (Hirose and Ogura, 2019) and epilepsy (Muñoz et al., 2017). Hamsters have been used to study infectious diseases: they are used to model certain cutaneous leishmaniasis (Mears et al., 2015) and prion diseases (Eckland et al., 2018), and recently they have increasingly been used as a model to study viral infections (Miao et al., 2019). For several viral diseases, hamsters offer certain advantages over mice as a disease model. For example, hamsters, but not mice, develop hemorrhagic fever-like disease following challenge with Ebola virus infection with symptoms very similar to those observed in humans (Ebihara et al., 2013). Syrian hamsters are the only rodents that develop a clinical disease similar to hantavirus pulmonary syndrome, and SARS-CoV infection results in severe respiratory disease in immunosuppressed hamsters (Safronetz et al., 2012; Schaecher et al., 2008). Hamsters are also a natural host for SARS-CoV-2, replicating the pathology and virus spread characteristics of the virus in human patients (Chan et al., 2020; Sia et al., 2020).

The rising popularity of the hamster model prompted the generation of much needed reagents and the development of genetically engineered hamster strains. The techniques to produce hamsters with specific gene knockout (KO) with the CRISPR/Cas9 system were pioneered by the laboratory of Zhongde Wang (Fan et al., 2014; Li et al., 2018a). As of today, results with six such strains have been reported: a signal transducer and activator of transcription 2 (*Stat2*) KO strain (Atkins et al., 2018; Gowen et al., 2017; Siddharthan et al., 2019; 2017; Toth et al., 2015); a *Kcnq1* potassium channel KO strain (Li et al., 2018b); a low-density lipoprotein receptor KO strain (Guo et al., 2018; He et al., 2019); a recombination activating gene 1 (*Rag1*) KO strain (Miao et al., 2018); a protocadherin-1 (*Pcdh1*) KO strain (Jangra et al., 2018); and a strain deleted for the tyrosinase gene (Hirose and Ogura, 2019).

Here, we report results with a new genetically engineered hamster strain in which the interleukin-2 receptor subunit gamma (common gamma chain; *Il2rg*) is inactivated. IL2RG is a shared component of the receptors for interleukins IL-2, IL-4, IL-7, IL-9, IL-15 and IL-21, which are required for the development of cells of the lymphoid lineage [T and B lymphocytes, natural killer (NK) cells and innate lymphoid cells] (Leonard et al., 2019). Defects in the *Il2rg* gene result in a profoundly immunocompromised status characterized by a dramatically decreased number of T lymphocytes, NK cells and non-functional B cells. With humans, the disease is called X-linked severe combined immunodeficiency (XSCID) (Noguchi et al., 1993). XSCID, in the absence of bone marrow transplantation, is generally lethal within the first year of life due to overwhelming infections (Heimall and Cowan, 2017). The standard therapy for XSCID is bone marrow transplantation; successful engraftment results in a functional immune system (with the occasional need for intravenous immunoglobulin therapy) and long-term survival of the recipient. Our model reflects the effects of the uncorrected disease and is useful for studying the multiple facets of this pleiotropic disease, with the caveat that there may be important differences between the human (especially the immature human) and hamster immune system. In comparison with other immunocompromised hamster strains, it will also be useful for studying what arms of the immune response are the most important in fighting off human pathogens that infect hamsters but not other laboratory animals, such as SARS-CoV-2 and human adenoviruses. As we learn more about the similarities and dissimilarities of the human and hamster immune systems, it is possible that these animals might be used as tools to study existing therapies, interactions of pathogen infection and host immunity, and develop novel approaches for the treatment of immunodeficiency diseases.

To study the effects of the *Il2rg* inactivation in our hamster strain, we chose to assess how knockout animals responded to infection with human adenovirus. Hamsters, as opposed to mice and rats, are permissive for the replication of human species C adenoviruses and develop a disease similar to that of humans after HAdV infection (Tollefson et al., 2017). Thus, hamsters are the most practical model for infection with replication-competent HAdV. The hamster model for the study of HAdV infections was developed in the William Wold laboratory and has been used to research the pathogenesis of HAdV, to test the efficacy of antiviral compounds, and to study oncolytic adenovirus vectors (reviewed in Wold and Toth, 2012, 2015; Wold et al., 2019). Human type 6 adenovirus (HAdV-C6) primarily causes respiratory infections; between 7% and 44% of people are seropositive for HAdV-C6 (Weaver et al., 2011). Recently, HAdV-C6 has been used for viral vector development (Weaver et al., 2011). In the hamster model, HAdV-C6 is the preferred challenge virus, because it is sequestered by non-permissive tissue macrophages to a lesser effect than HAdV-C5, resulting in increased virus replication and lower LD_50_ compared to HAdV-C5 (Tollefson et al., 2017). In our studies, we administered HAdV-C6 intravenously, aiming to mimic disseminated HAdV infection seen with immunocompromised patients. Adenovirus infections of immunocompetent adults are generally not thought to be dangerous, inasmuch as the immune system effectively eliminates the virus and limits pathology (Wold and Ison, 2013). Recently, with the widespread use of immunosuppressive regimes in organ transplantation, the size of the immunocompromised population has grown. With this group of patients, the frequency and severity of systemic adenovirus infections have increased (Lion, 2014). Species C HAdV (HAdV-C6 belongs to this group) are isolated most frequently from these patients. During systemic infection, these primarily respiratory viruses cause disseminated disease involving multiple organs, among them the lung, the liver and the gastrointestinal tract (Feghoul et al., 2015). In our experiments, we chose to monitor the liver of these organs, as the liver is the main target of systemic HAdV infection in the hamster model (Tollefson et al., 2017). An animal model mimicking human XSCID will be invaluable to study adenovirus (and other viral) infections of immunocompromised patients. Furthermore, *Il2rg* KO hamsters will complement other existing XSCID rodent models and provide new insight into the human condition.

## RESULTS

### Generation of *Il2rg* KO hamsters

**Fig. 1.**
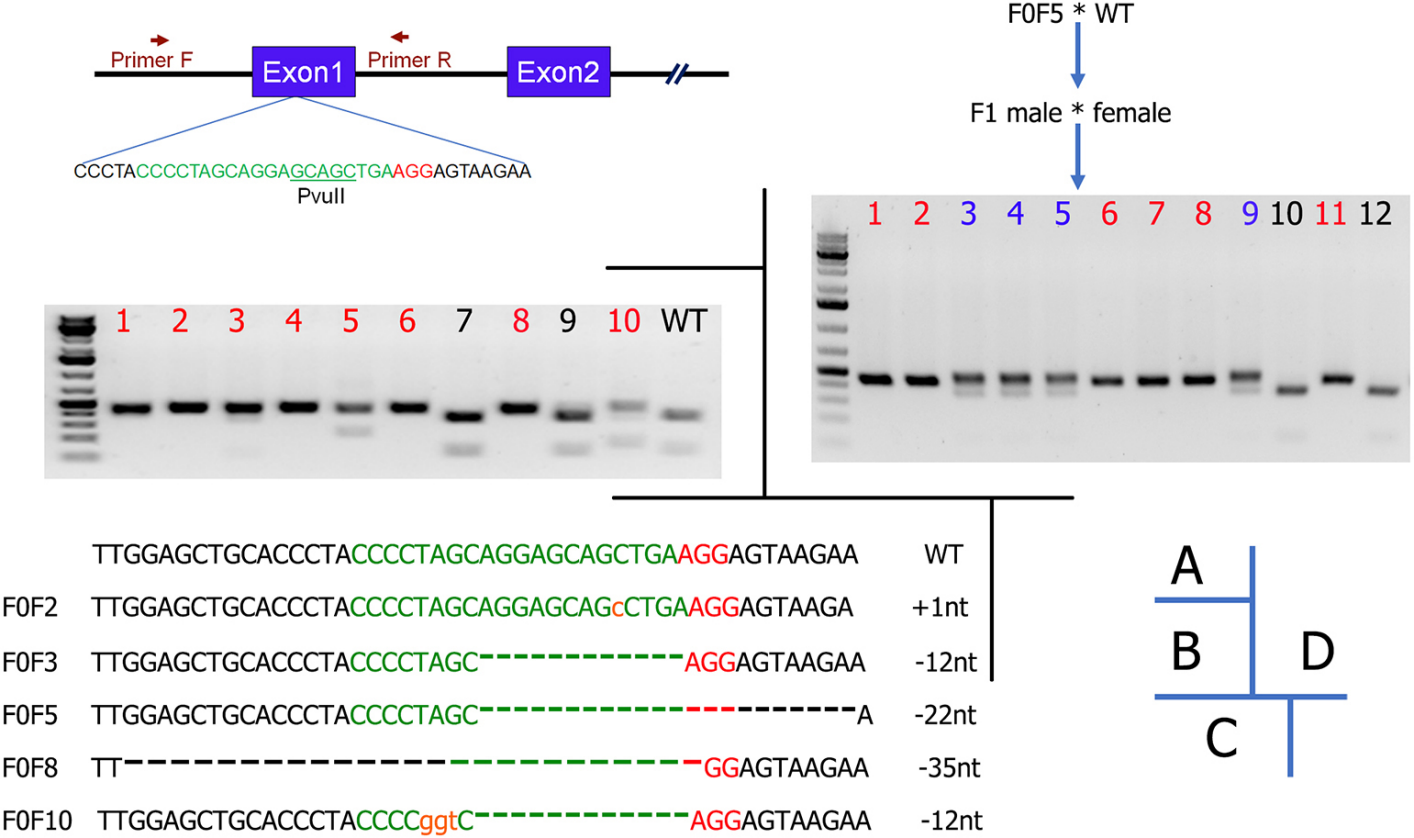
***Il2rg*** **sgRNA design and genotyping.** (A) Diagram of the genomic region containing the first two exons of *Il2rg*, sgRNA design, and the PCR primers and Pvull restriction site used for PCR-RFLP genotyping assays. The sgRNA sequence is depicted in green and the protospacer adjacent motif (PAM) in red. (B) PCR-RFLP identified eight (in red) out of ten pups carrying targeted indels. (C) Different indels identified by Sanger sequencing the TA clones, including three frameshifts: 1 bp insertion (+1nt; F0F2), 22 bp deletion (−22nt; F0F5) and 35 bp deletion (−35nt; F0F8). In-frame deletions were also identified (F0F3 and F0F10). (D) Establishment of Syrian hamster *Il2rg* KO colony by crossing F0F5 with wild type (WT), followed by crossing their F1 littermates. Pups corresponding to the lanes marked in red are homozygous KO.

We employed the CRISPR/Cas9-mediated genetic engineering techniques that we established in Syrian hamsters to genetically inactivate the hamster *Il2rg* (Fig. 1A). An sgRNA was designed to target exon 1 of *Il2rg* based on the on-target and off-target scores calculated using the sgRNA design tools from both Benchling (www.benchling.com/crispr/) and CRISPRdirect (https://crispr.dbcls.jp/). The sgRNA was synthesized using a GeneArt transcript kit (Thermo Fisher, A29377) and assembled into the sgRNA/Cas9 ribonucleoprotein (RNP) complex for pronuclear injection into hamster embryos. Following the transfer of 143 pronuclear-injected embryos into five pseudopregnant females, ten pups were produced (Table 1). Genotyping analysis with the PCR-restriction fragment length polymorphism (RFLP) assay identified eight pups carrying targeted indel mutations (Fig. 1B; PCR primers are listed in Table 1). To reveal the nature of the indels, PCR products produced from the genomic DNA isolated from the ear punches of five selected pups, F0F2, F0F3, F0F5, F0F8 and F0F10, were subcloned into TA vectors that were subsequently subjected to Sanger sequencing (Fig. 1C).

**Table 1.**
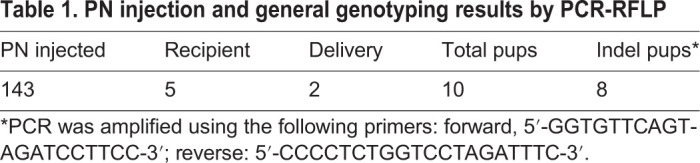
**PN injection and general genotyping results by PCR-RFLP**

To validate the specificity of the sgRNA used to generate the KO animals, we conducted off-target analyses by using the genomic DNA isolated from F0 animals. BLAST searches of the Syrian hamster genome (www.ncbi.nlm.nih.gov/genome/11998) with the sgRNA sequence identified six potential off-target sites (with the highest homology to the sgRNA sequence) (Table 2). The potential off-targeting sites, OT1-6, were analyzed with PCR-RFLP assays followed by Sanger sequencing of the PCR products. Our analyses showed that no off-targeting event occurred in the analyzed animals (data not shown), indicating that the sgRNA is highly specific, as predicted by the two online sgRNA design tools [Benchling and CRISPOR (Crispor.tefor.net)]. Therefore, we concluded that *Il2rg* had been successfully targeted. To establish an *Il2rg* KO colony, we chose animal F0F5, which carries a 22-bp frameshift deletion (Fig. 1C), as the F0 founder and bred it to wild-type animals to produce F1 animals. We then generated homozygous KO animals by crossing littermates of the heterozygous KO F1 animals. As shown in Fig. 1D, F2 pups 1, 2, 6, 7, 8 and 11 were homozygous KOs, 3, 4, 5 and 9 were heterozygous, whereas 10 and 12 were wild type. We then established a breeding colony by using the homozygous KO F2 pups as breeders. All the experiments described below used the animals produced from this breeding colony.

**Table 2.**
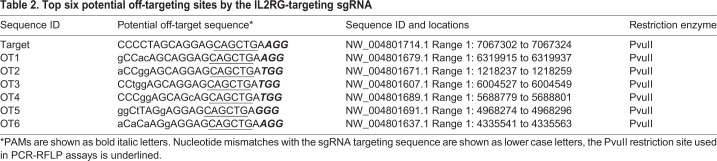
**Top six potential off-targeting sites by the IL2RG-targeting sgRNA**

### *Il2rg* KO hamsters have a severe defect in the development of lymphocytes

**Fig. 2.**
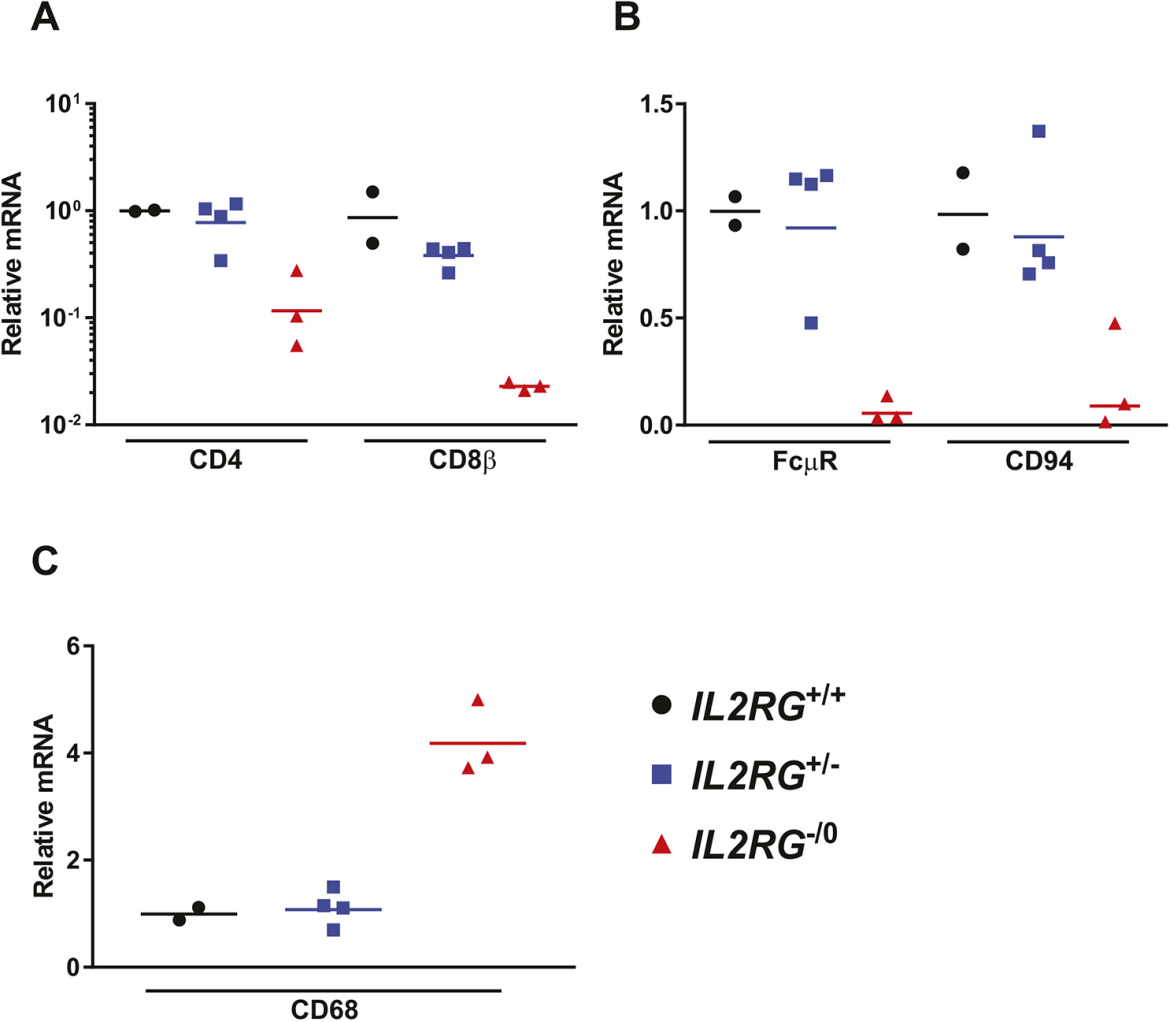
***Il2rg* KO (*Il2rg*^−/0^) hamsters have fewer T and B lymphocytes and natural killer cells in their spleen compared to their wild-type (*Il2rg*^+/+^) and heterozygous (*Il2rg*^+/−^) littermates.** (A-C) The graphs show relative levels of mRNAs, as determined by RT-qPCR assays. For each transcript, the mean of the values for wild-type animals was established as the baseline (‘1’ on the graphs). For this and subsequent similar graphs, symbols depict data from individual animals, and the horizontal bar signifies the geometric mean. *Il2rg*^+/+^, *n*=2; *Il2rg*^+/−^, *n*=4; *Il2rg*^−/0^, *n*=3.

To investigate the effect of the disruption of the *Il2rg* gene on the development of cells of lymphocytic lineage, we compared the expression of mRNAs for several marker genes that are key representatives of lymphocyte populations in the spleen of homozygous and heterozygous *Il2rg* KO and wild-type littermates. To assess the abundance of CD4^+^ T cells, CD8^+^ T cells, B cells and NK cells, we quantified the mRNAs for CD4, CD8β, Fc mu receptor (FcµR) and CD94, respectively. We found that in wild-type littermates, the expression levels of these transcripts were similar to those seen in wild-type hamsters in previous experiments (data not shown). However, mRNA levels for these markers were significantly lower in the homozygous *Il2rg* KO hamsters, suggesting lower lymphocyte numbers in the spleen (Fig. 2A,B). Conversely, the relative abundance of CD68-expressing cells (i.e. macrophages and dendritic cells) in the spleen of homozygous *Il2rg* KO hamsters was higher than in the spleen of wild-type animals (Fig. 2C). Data obtained with heterozygous *Il2rg* KO hamsters were identical to that of wild-type animals, indicating that haplosufficiency allows the development of these lymphocytic lineages.

### After intravenous infection, HAdV-C6 replicates more and causes more pathology in the liver of *Il2rg* KO hamsters

**Fig. 3.**
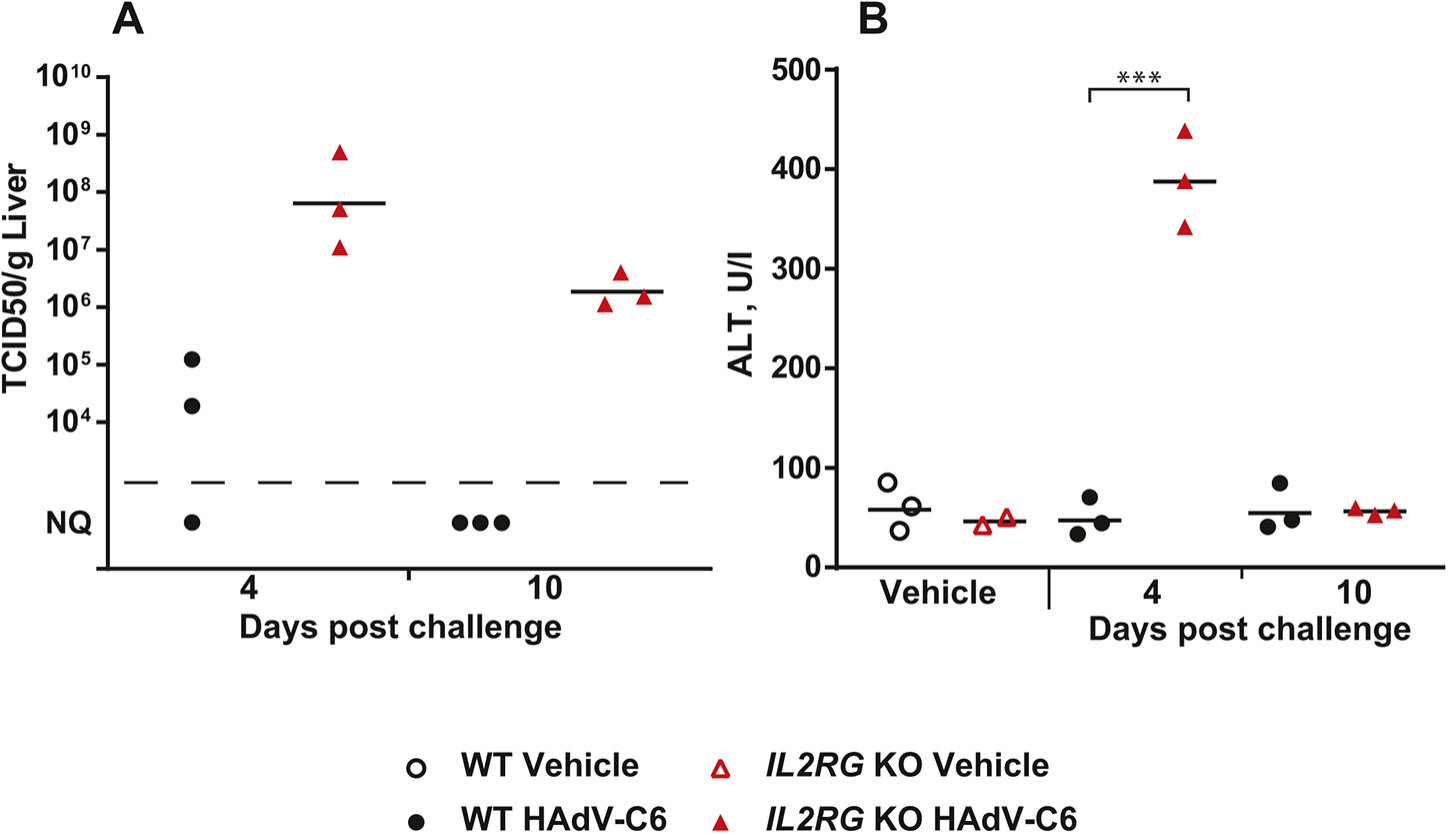
**Intravenous injection with HAdV-C6 results in higher virus burden in the liver and causes more liver pathology with *Il2rg* KO hamsters than with wild-type animals.** (A) At both 4 and 10 days post challenge, *Il2rg* KO hamsters have more infectious virus in their liver. (B) At 4 days post challenge, ALT levels are higher in the serum of *Il2rg* KO hamsters than in wild-type (WT) animals (B). Wild-type vehicle, *n*=3; *Il2rg* KO vehicle, *n*=2; wild-type HAdV-C6, *n*=6; *Il2rg* KO HAdV-C6, *n*=6. NQ, not quantifiable; statistical significance cannot be calculated. ***P<0.001 (Mann–Whitney U-test).

To assess whether the defect in lymphocyte development in the *Il2rg* KO hamsters impairs the response to viral infections, we challenged these animals with an intravenous infection of HAdV-C6 (Tollefson et al., 2017). At 4 days post challenge, the *Il2rg* KO hamsters had significantly higher peak viral titers, with 1000-fold higher infectious virus burden in the liver compared to wild-type animals, indicating enhanced replication in the former (Fig. 3A). Furthermore, clearance of the virus was impaired in the *Il2rg* KO hamsters, inasmuch as significant amounts of infectious virus were found in the liver of these animals at 10 days post challenge, whereas the wild-type hamsters had largely eliminated the virus by this time (Fig. 3A). This increased and prolonged virus replication in the liver of *Il2rg* KO hamsters resulted in exacerbated liver pathology, as indicated by a significant increase in alanine aminotransferase (ALT) (Fig. 3B).

### Lymphocytes fail to respond to infection with HAdV-C6 in *Il2rg* KO hamsters

**Fig. 4.**
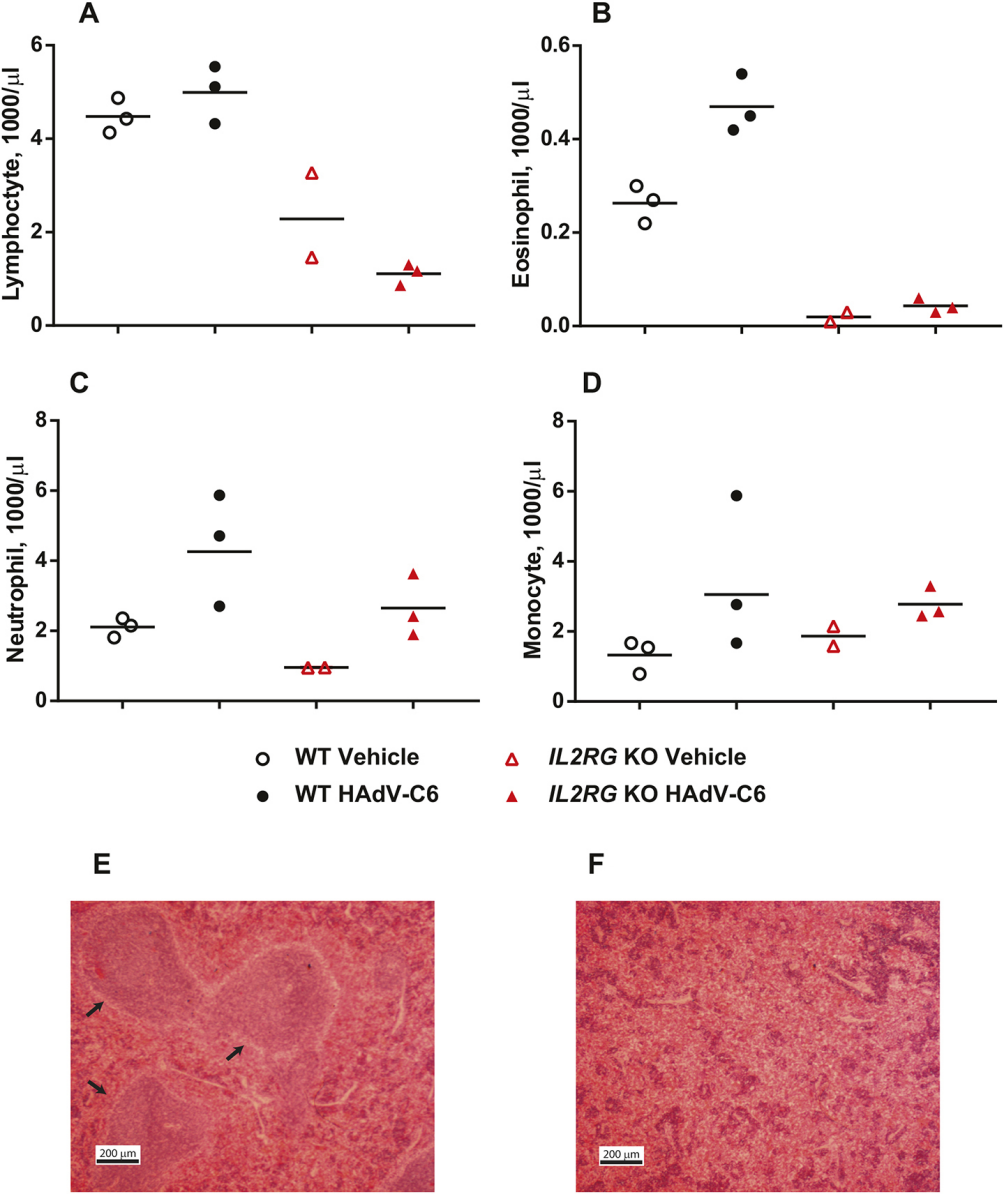
**The lymphocyte compartment does not expand in response to HAdV-C6 infection in *Il2rg* KO hamsters like it does in wild-type animals.** (A-F) At 10 days post challenge, the number of all leukocyte subsets assayed for lymphocytes (A), eosinophils (B), neutrophils (C) and monocytes (D) was elevated for the wild-type (WT) hamsters. The number of lymphocytes (A) and eosinophils (B) in the blood remains low for the *Il2rg* KO animals. No differences were seen in the abundance of neutrophils and monocytes. Wild-type vehicle, *n*=3; *Il2rg* KO vehicle, *n*=2; wild-type HAdV-C6, *n*=3; *Il2rg* KO HAdV-C6, *n*=3. Large germinal centers (arrows) were observed in the spleen of wild-type hamsters (E); however, the spleen of the *Il2rg* KO hamsters was disorganized (F). Representative images of three animals for each group are shown.

With wild-type hamsters, lymphocyte numbers in the peripheral blood increased (Fig. 4A) and germinal centers formed in the spleen (Fig. 4E) at 10 days post challenge. Conversely, no clonal expansion was observed for *Il2rg* KO hamsters, and classical spleen architecture was disorganized (Fig. 4E,F). In addition, eosinophil granulocytes, another leukocyte type that is dependent on IL-7 during development, also failed to expand with the *Il2rg* KO animals, and there were marginally lower numbers of neutrophil granulocytes in the blood of *Il2rg* KO hamsters (Fig. 4B,C). After challenge with HAdV-C6, there was a similar increase in the numbers of circulating monocytes in wild-type and *Il2rg* KO hamsters (Fig. 4D).

**Fig. 5.**
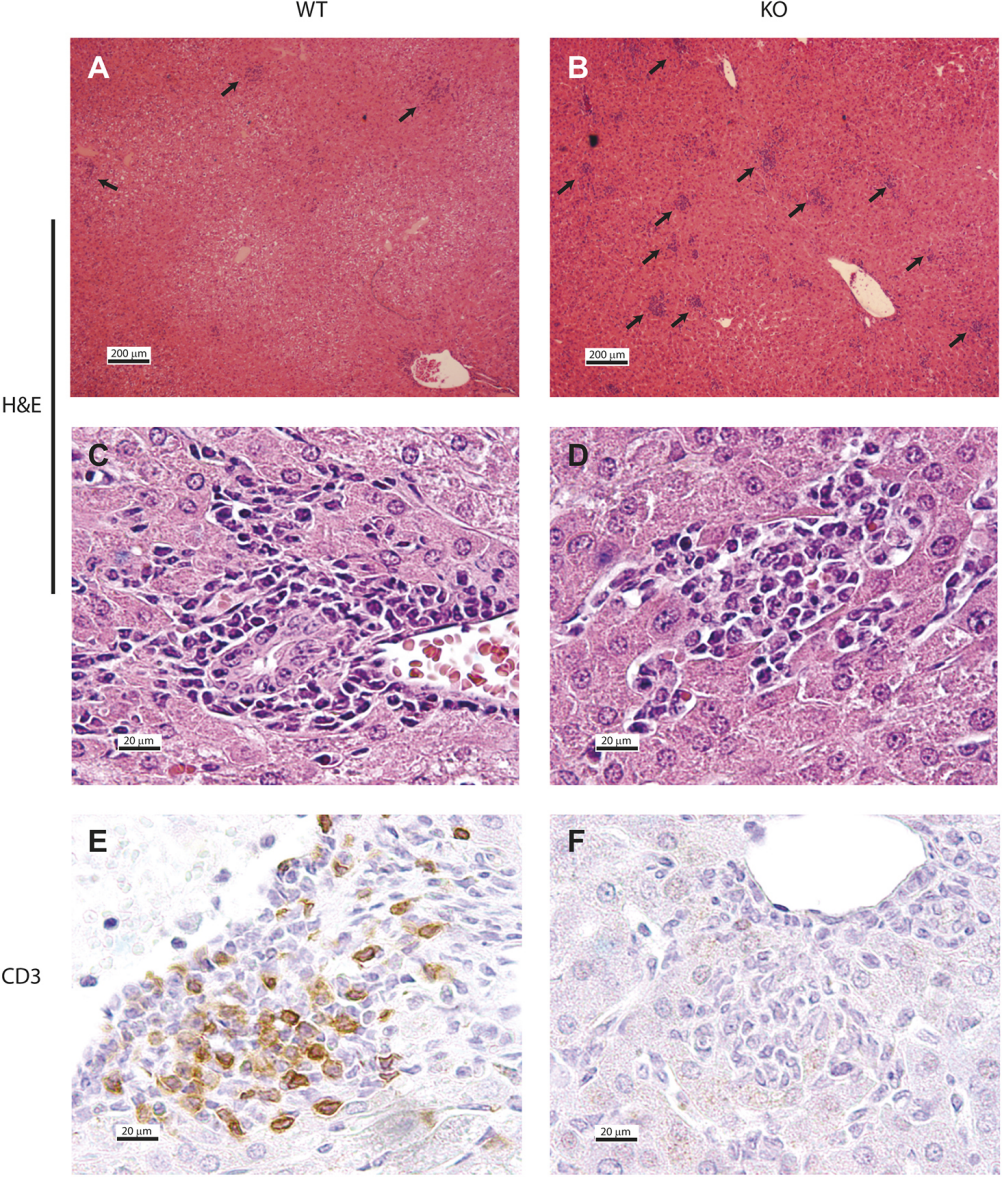
**The immune infiltration is different in the liver of *Il2rg* KO hamsters than in the liver of wild-type animals.** (A-F) *Il2rg* KO animals have more infiltrating cells (arrows) in their liver than wild-type (WT) animals (A,B). The foci consist of mononuclear cells for both strains (C,D); however, the foci of *Il2rg* KO hamsters do not contain CD3^+^ cells (E,F). Images A-D show Hematoxylin-Eosin (H&E) staining, whereas E and F depict immunohistochemistry for CD3. Representative images of three animals for each group are shown.

The failure of lymphocytes to react to HAdV-C6 infection is also reflected in the immune infiltration of the liver. Although *Il2rg* KO hamsters had more infiltrating cell foci in the liver than wild-type hamsters (Fig. 5A-D), the nature of the infiltration was different. Although a sizable portion of the infiltrating cells in wild-type hamsters stained positive for CD3, indicating that these were T lymphocytes or NKT cells, the foci in the livers of *Il2rg* KO hamsters were completely void of CD3^+^ cells (Fig. 5E,F).

**Fig. 6.**
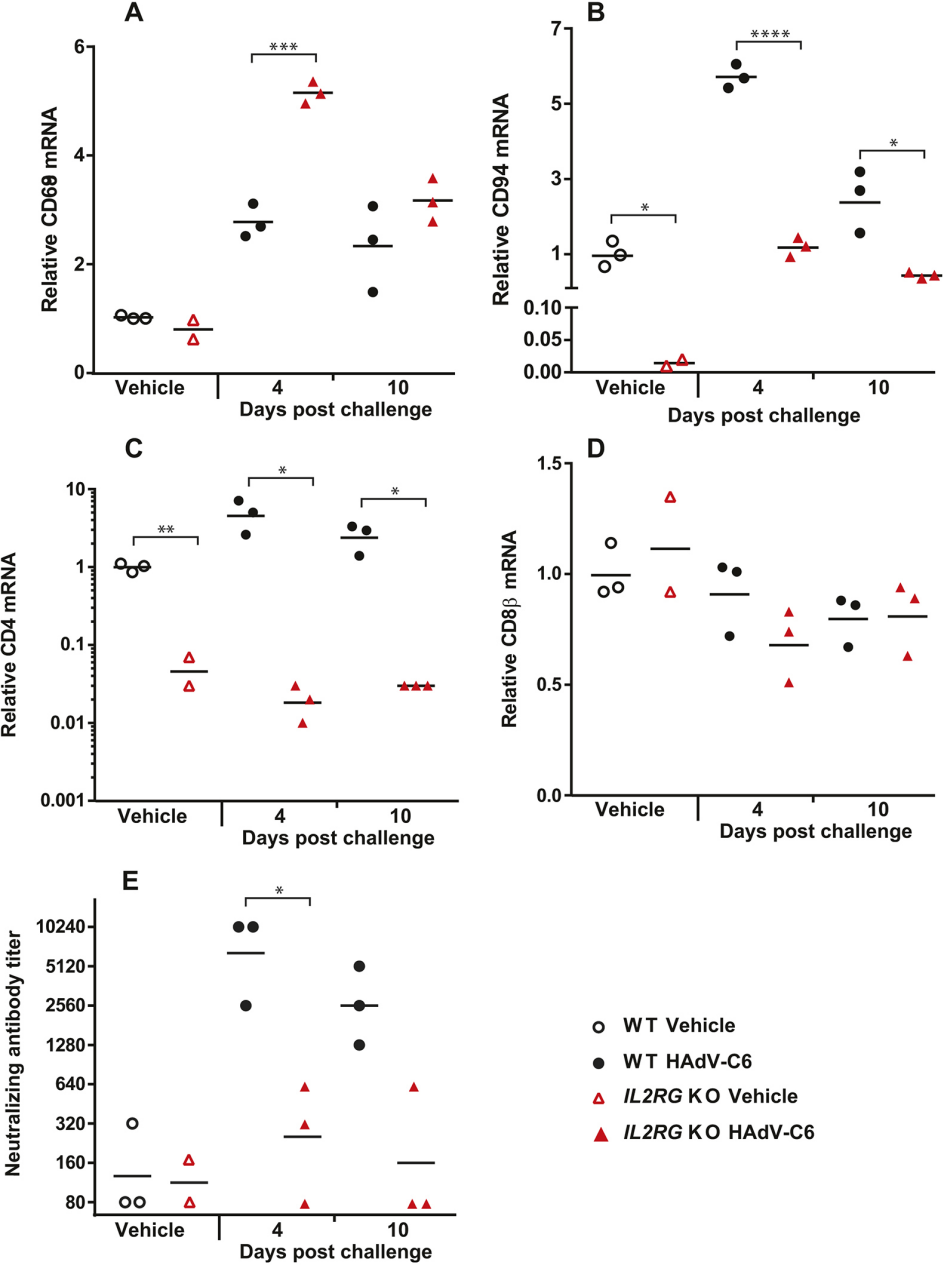
**In response to HAdV-C6 infection, macrophages dominate the cells infiltrating the liver in *Il2rg* KO hamsters, whereas the lymphocyte-dependent immune response is impaired.** (A-D) Compared to wild-type (WT) hamsters, there was significantly more macrophage infiltration in the liver of *Il2rg* KO hamsters (A). Conversely, there was less NK cell (B) and CD4^+^ lymphocyte (C) infiltration in the liver of both naïve and HAdV-C6-infected *Il2rg* KO animals, whereas CD8^+^ lymphocytes did not infiltrate the livers of either wild-type or *Il2rg* KO hamsters in response to HAdV-C6 infection (D). (E) *Il2rg* KO hamsters generated only marginal levels of neutralizing antibodies. Wild-type vehicle, *n*=3; *Il2rg* KO vehicle, *n*=2; wild-type HAdV-C6, *n*=6; *Il2rg* KO HAdV-C6, *n*=6. *P<0.05; **P<0.01; ***P<0.001; ****P<0.0001 (Mann–Whitney U-test).

To further characterize the infiltrating cells in the liver, we analyzed the abundance of immune cell-specific transcripts in vehicle-treated and HAdV-C6-infected hamsters of both strains. We found a significant increase of a macrophage- and dendritic cell (DC)-specific mRNA (*Cd68*) in the livers of HAdV-C6-infected *Il2rg* KO hamsters compared to wild-type animals (Fig. 6A), and, as expected, significantly lower expression of T lymphocyte- (Fig. 6C,D) and NK cell-specific (Fig. 6B) transcripts. However, there was a notable ∼100-fold increase in the amount of NK cell-specific mRNA (*Cd94*) in response to HAdV-C6 infection in the livers of *Il2rg* KO hamsters compared to mock-infected animals of the same strain (Fig. 6B). Nonetheless, even with this increase, the CD94 transcript level of HAdV-C6-infected *Il2rg* KO hamsters barely reached the steady state level measured in mock-infected wild-type animals (Fig. 6B).

**Fig. 7.**
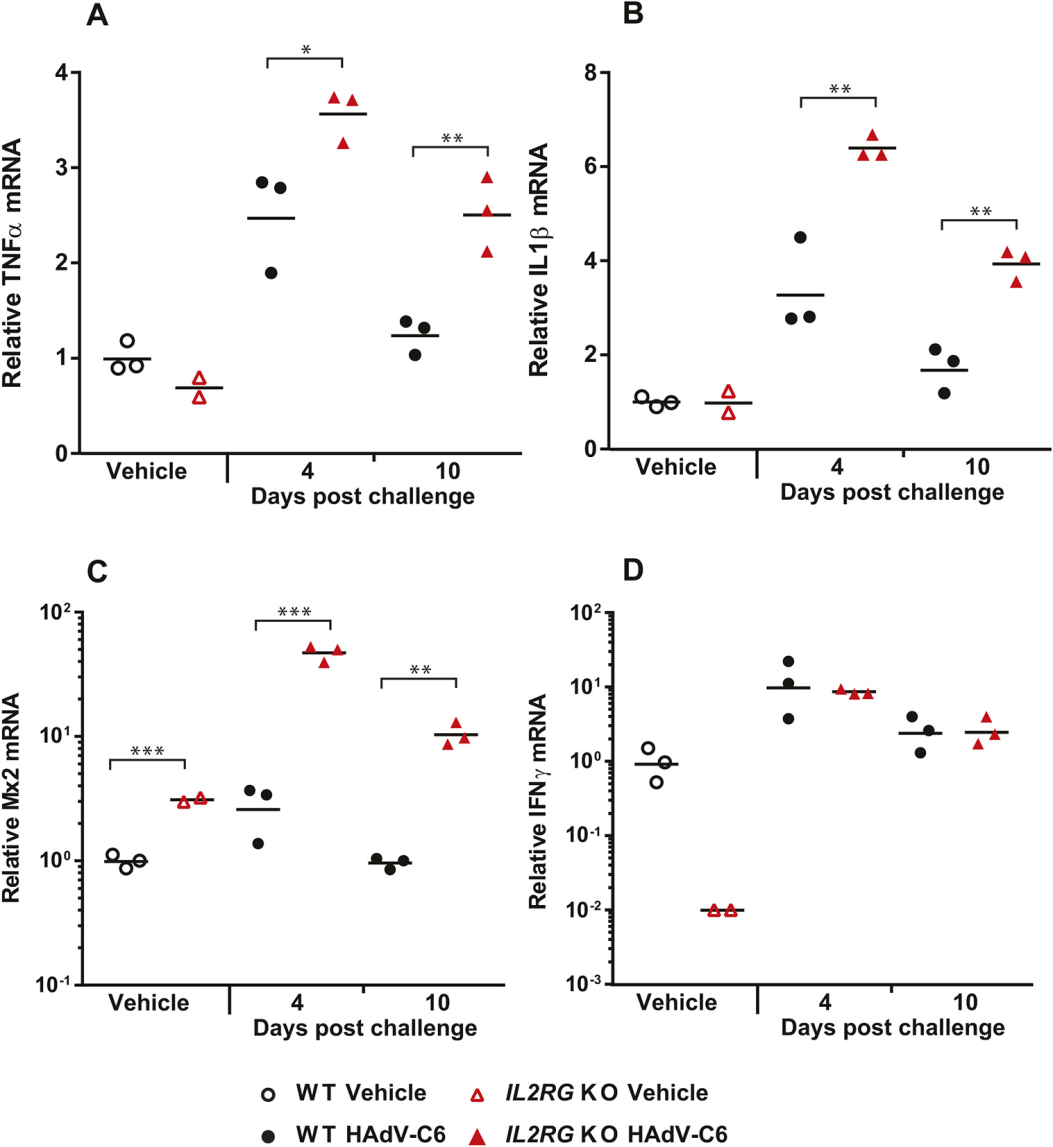
**Following HAdV-C6 infection, the inflammatory** **cytokine response and the type I IFN response is elevated in *Il2rg* KO hamsters compared to wild type, whereas the type II IFN response is normal.** (A-C) The mRNA levels for TNFα (A) and IL1β (B) are higher in the liver of *Il2rg* KO hamsters than for wild-type (WT) hamsters, as are the mRNA levels for *Mx2*, a type I ISG (C). (D) Naïve *Il2rg* KO hamsters produce less IFNγ than wild-type hamsters in their liver; however, they can induce production of the cytokine at similar levels to wild type after infection. Wild-type vehicle, *n*=3; *Il2rg* KO vehicle, *n*=2; wild-type HAdV-C6, *n*=6; *Il2rg* KO HAdV-C6, *n*=6; *P<0.05; **P<0.01; ***P<0.001 (Mann–Whitney U-test).

To assess the functionality of the adaptive immune response, we first tested the ability of the animals to produce neutralizing antibodies (NAbs). The *Il2rg* KO hamsters had only marginal levels of NAbs in the serum, whereas the wild-type hamsters produced high amounts (Fig. 6E), confirming that similar to T lymphocytes and NK cells, the functional reaction of B lymphocytes to virus infection is also impaired. When analyzing the changes in expression levels of certain cytokine-specific mRNAs in the liver of HAdV-C6-infected hamsters, we found that the expression of *Tnfa* and *Il1b* transcripts were significantly higher in *Il2rg* KO hamsters than in wild-type ones (Fig. 7A,B); these findings are consistent with enhanced macrophage and DC infiltration with the *Il2rg* KO hamsters. The expression of *Mx2*, a type I IFN-stimulated gene (ISG), was also higher in the *Il2rg* KO hamsters compared to the wild-type ones (Fig. 7C). Unexpectedly, the expression levels of IFNγ in the liver of *Il2rg* KO hamsters were similar to those for wild-type animals (Fig. 7D).

## DISCUSSION

The Syrian hamster is an extremely important animal model for several human infectious diseases either because a pathogen replicates well in hamsters or because the pathogenesis in these animals resembles that seen in human patients (Miao et al., 2019). Furthermore, certain human cytokines, such as IFNα, GM-CSF and IL-12, are functional in hamsters but not in mice, allowing for the testing of human therapeutics in hamsters (Bortolanza et al., 2009; Cho et al., 2006; Shashkova et al., 2007). In spite of the importance of this animal model, the development of genetic hamster models has been lagging behind other more popular animal models. Previously, we reported on the generation and characterization of *Stat2*- and partially *Rag1*-deficient hamster strains. We generated the *Il2rg* KO hamster strain with the intention of gaining further insight into the immune response in these animals.

The model animal for studying the effect of *IL2RG* deletion has been the *Il2rg* KO mouse (Cao et al., 1995). This animal largely recapitulates the characteristics of XSCID patients, and we report similar findings for the *Il2rg* KO hamsters. The lymphoid compartment was severely reduced in numbers and functionality. *Il2rg* KO hamsters raised a subpar immune response to HAdV-C6 infection, with major defects in both the humoral and cellular arms of the immune system. The increase in virus replication with *Il2rg* KO hamsters was similar to that seen with *Stat2* KO animals; at 4 and 3 days, respectively post challenge, we observed ∼1000-fold higher virus burden in the livers of both KO strains than in wild-type hamsters [Fig. 3A; Toth et al. (2015)]. Notably, in the *Il2rg* KO animals at 4 days post challenge, the type I IFN-stimulated gene *Mx2* was activated, indicating that the type I IFN pathway is intact. In fact, the expression of Mx2 mRNA was increased in *Il2rg* KO hamsters compared to wild-type hamsters; we suspect that this is because of the increased virus load in the *Il2rg* KO animals. This suggests that in hamsters, both type I IFN and cells of lymphoid lineage (possibly NK cells) are important in the early defense against HAdV infection.

One important difference between human XSCID patients and *Il2rg* KO mice is that B cells are absent in *Il2rg* KO mice, whereas B cells are present in normal or even elevated numbers in XSCID patients but are non-functional (Cao et al., 1995; Noguchi et al., 1993). This is thought to be because of the requirement of IL-7 as a growth factor for pre-B cells in mice but not in humans (Puel et al., 1998; von Freeden-Jeffry et al., 1995). In a recently reported *Il2rg*-deficient rat strain, B cell numbers were normal but the cells were not functional, indicating that the impact of the *Il2rg* inactivation on the early developmental steps of B cells varies from genus to genus. A comparative approach between mice, rats and hamsters may lead to an improved understanding of primary B cell deficiencies in humans (Ménoret et al., 2018), and such an approach may be beneficial for evaluating therapeutics. It seems that in this respect, hamsters are similar to mice, as we observed greatly reduced numbers of B cells in the spleen of *Il2rg* KO hamsters (Fig. 2B). Nevertheless, hamsters (and mice and rats) resemble humans in that *Il2rg* inactivation disrupted B cell function; no germinal centers formed in the spleen after virus infection and only a marginal amount of neutralizing antibody was produced after virus infection (Fig 4E,F; Fig. 6E).

Loss of function in *IL2RG* prevents signaling through the IL-15 receptor. As IL-15 is an important survival factor for NK cells, XSCID patients and *Il2rg* KO mice and rats have greatly reduced numbers of NK cells (Cao et al., 1995; Ménoret et al., 2018; Noguchi et al., 1993). We detected a similar decrease in the spleen and liver of *Il2rg* KO hamsters, and there were ∼sixfold more infiltrating NK cells in the liver of wild-type hamsters at this time (Fig 2B; Fig. 6B). As NK cells respond very early to virus infection (Ali et al., 2019), we reason that this loss of NK cells is responsible for the increased virus replication in the liver of *Il2rg* KO hamsters at 4 days post challenge. However, the absence of NK cells was not complete: at 4 days post challenge with HAdV-C6, there was a ∼100-fold increase in the number of infiltrating NK cells in the liver (Fig. 6B). This residual NK cell population might be responsible for the production of IFNγ after infection with HAdV-C6, which was at par with that in the liver of wild-type hamsters (Fig. 7D). Alternatively, another cell type might account for this phenomenon. It has been reported that both human and mouse macrophages can produce IFNγ after stimulation with IL-12 and IL-18 (Darwich et al., 2009; Munder et al., 1998). Credence is given to this scenario by the observation that with the *Il2rg* KO hamsters, a large number of macrophages infiltrated the liver after HAdV-C6 infection (Fig 5B,D; Fig. 6A), and the expression of mRNA for TNFα and IL-1β (cytokines expressed by macrophages) is increased compared to wild-type hamsters (Fig. 7A,B). Another cell type that might contribute to IFNγ production in the liver of HAdV-C6-infected hamster is neutrophil granulocytes, which were shown to express IFNγ when stimulated by IL-12 (Ethuin et al., 2004). Clearly, this phenomenon needs to be further investigated.

All things considered, *Il2rg* KO Syrian hamsters provide a new tool for studying the immunological disorders caused by IL2RG deficiency in humans and the associated infectious diseases in this important model animal. This novel *Il2rg* KO Syrian hamster line might also be useful for studying cancer immunology, as well as being used as a host for human stem cell and cancer cell transplantation research.

## MATERIALS AND METHODS

### Cells and viruses

A549 human lung adenocarcinoma cells were purchased from the American Type Culture Collection (ATCC), whereas HEK293 human embryonic kidney cells were purchased from Microbix. Both cell lines were cultured in Dulbecco's modified Eagle's medium (Sigma-Aldrich) with 10% fetal bovine serum at 37°C. A wild-type human HAdV-C6 isolate (VR-6; Tonsil 99) was purchased from ATCC and cultured and purified as described by Tollefson et al. (2007). The titer of the virus stocks was determined by plaque assay.

### Animals

The *Il2rg* KO hamsters were generated and bred at the Laboratory Animal Research Center at Utah State University. Wild-type Syrian hamsters were purchased from Envigo at ∼100 g body weight. All animals were aged between 5 and 8 weeks old at the time of infection. All studies were approved by the Institutional Animal Care and Use Committee of Saint Louis University and were conducted according to federal and institutional regulations.

### *In vivo* infection of HAdV-C6 in wild-type and *Il2rg* KO Syrian hamsters

In the experiment, three groups of uninfected Syrian hamsters, including *Il2rg* KO male (*Il2rg*^−/0^, *n*=3), heterozygous female (*Il2rg*^+/−^, *n*=4) and wild-type male and female (*Il2rg*^+/+^, *n*=2) littermates, were used to characterize and validate the phenotypic defect of *Il2rg* gene inactivation. Spleen samples from each animal were collected and spleen RNAs were extracted to quantify the transcripts corresponding to specific innate and adaptive immune cell populations by RT-qPCR.

In the experiment to determine the effect of common gamma chain gene knockout on the replication and pathogenicity of human type 6 adenovirus in Syrian hamsters, two groups of hamsters were established; one with *Il2rg* KO animals (*n*=8) and the other with wild type (*n*=9). The animals were anesthetized with a ketamine/xylazine mixture (47.5 mg/kg ketamine; 3.5 mg/kg xylazine), and PBS or HAdV-C6 was injected intravenously (in 200 µl volume via the jugular vein). Three hamsters in the wild-type group and two hamsters in the *Il2rg* KO group received vehicle (PBS), whereas the remaining six hamsters in each group were injected with 1×10^10^ plaque forming units (PFU)/kg of HAdV-C6. Three HAdV-C6-infected hamsters from each group were sacrificed at 4 days after challenge; the remaining three HAdV-C6-infected hamsters from each group were sacrificed at 10 days after challenge. The body weights and signs of morbidity of the animals were recorded daily. For all animals, blood was collected for white blood cell count, sera were assayed for alanine transaminase levels (Advanced Veterinary Laboratory) and serum neutralizing antibody levels. Liver samples were collected for determining virus burden and levels of several immune-related RNAs (such as cytokines and cellular markers) using RT-qPCR. For all animals, portions of liver tissue were preserved in formalin for histopathological and immunohistochemical staining.

### Necropsy, histopathology and clinical pathology

At necropsy, the animals were bled out and liver samples were collected. Virus was extracted from the liver and was quantified by the 50% tissue culture infectious dose (TCID_50_) assay in HEK293 cells as described previously (Toth et al., 2008). A portion of the collected tissues was preserved in formalin and processed for histopathology (Seventh Wave Laboratories). Immunohistochemical staining was performed by the Histopathology and Tissue Shared Resource at Georgetown University, using a 1:200 dilution of the CD3-ε antibody (M-20) (Santa Cruz Biotechnology, sc-1127) to stain for hamster CD3 proteins. Sera transaminase levels were determined by Advanced Veterinary Laboratories, whereas hematological values were assessed using an Idexx ProCyte DX hematology analyzer.

### Determining the relative mRNA abundance for immune-related genes using RT-qPCR

Total RNA from liver and spleen was extracted by homogenizing a fraction of collected tissues in RLT lysis buffer (Qiagen) and then extracting the RNA using an RNeasy Mini Kit (Qiagen). All RNA samples were treated with RNase-free DNase followed by RNA cleanup to eliminate DNA contamination. The RNA yield was determined using a NanoDrop-2000 spectrophotometer.

For RT-qPCR, 1.5 μg to 2 μg of RNA and 50 pM of oligo(dT) primer were used for the reverse transcription using the High-Capacity cDNA Reverse Transcription Kit (Applied Biosystems). SYBR-green-based qPCR was used to specifically detect target gene mRNA (Applied Biosystems). Primer sequences for *Mx2*, *Il1b*, *Ifng*, *Rpl18* (housekeeping gene), *Cd68* (macrophage and dendritic cell marker), *Cd94* (NK cell marker), *Cd4* (CD4T cells), *Cd8b* (CD8T cells) and IgM receptor *FcμR* (B cells) were previously described (Miao et al., 2018; Ying et al., 2018; Zivcec et al., 2011). The data were analyzed using the ΔΔCt method. Housekeeping gene *Rpl18* was used as an endogenous control for normalization. The final value is displayed as the relative fold change between the HAdV-C6-infected and vehicle-treated hamsters.

### Determining the anti-HAdV-C6 neutralizing antibody (NAb) titers in the sera

Anti HAdV-C6 NAb in the sera were quantified as described previously (Toth et al., 2015). Briefly, sera samples were inactivated by heat treatment at 56°C for 30 min. One hundred PFU of HAdV-C6 were incubated with twofold serial dilutions of sera samples at 37°C for 1 h. Following incubation, A549 cells were infected with the virus-serum mixture, and the NAb titer was calculated as the reciprocal dilution causing 50% inhibition of viral cytopathic effect.

### Statistical analysis

Statistical analysis was performed using GraphPad Prism 7 (GraphPad Software). For sera transaminase levels, virus burden in various organs, and for mRNA levels, the variance of samples in all groups was calculated using the Kruskal–Wallis test, and comparison between groups was performed using the two-tailed Mann–Whitney U test. *P*≤0.05 was considered significant.

### Ethics statement

All animal studies were approved by the Institutional Animal Care and Use Committee of Saint Louis University (protocol 2015). The studies were conducted according to the regulations of Animal Welfare Act, the Public Health Service Policy on Humane Care and Use of Laboratory Animals, and according to the recommendations of the Guide for the Care and Use of Laboratory Animals**.**
